# Special Issue “Circulating Non-Coding RNAs as Diagnostic and Prognostic Markers of Human Diseases”

**DOI:** 10.3390/ijms26199448

**Published:** 2025-09-27

**Authors:** Kyriacos Felekkis, Christos Papaneophytou

**Affiliations:** 1 Department of Life Sciences, School of Life and Health Sciences, University of Nicosia, Nicosia 2417, Cyprus; felekkis.k@unic.ac.cy; 2 Non-Coding RNA Research Laboratory, School of Life and Health Sciences, University of Nicosia, Nicosia 2417, Cyprus

Biomarkers are measurable biological molecules that reflect normal physiological processes, pathological states, or responses to environmental exposures and therapeutic interventions [1]. Their clinical significance resides in their capacity to facilitate early disease detection, guide diagnostic decision making, predict patient prognosis, and monitor therapeutic responses. Over the past decade, research into non-coding RNAs (ncRNAs), including microRNAs (miRNAs), long non-coding RNAs (lncRNAs), circular RNAs (circRNAs), and PIWI-interacting RNAs (piRNAs), has expanded exponentially, revealing robust associations with diverse pathological conditions spanning cancer, cardiovascular disorders, and autoimmune diseases [2]. This growing field has witnessed substantial progress in leveraging ncRNAs as diagnostic and prognostic biomarkers, thereby establishing new paradigms for precision patient management from early disease detection through monitoring of recurrence and therapeutic response [3].

Furthermore, circulating ncRNAs (c-ncRNAs) have emerged as particularly compelling biomarker candidates due to their remarkable stability in biological fluids, ready accessibility in samples such as blood and urine, and distinctive disease- and tissue-specific expression profiles [4]. Beyond their favorable detection characteristics, c-ncRNAs function as critical regulators of fundamental cellular processes, including proliferation, apoptosis, and cell cycle progression. Consequently, they participate in disease pathogenesis through multiple mechanisms, serving variously as causative agents, modulators of pathological processes, or downstream effectors of disease states. The translational potential of c-ncRNAs as non-invasive, liquid-biopsy-based biomarkers is further enhanced by their capacity to provide mechanistic insights into disease pathophysiology and to dynamically reflect therapeutic responses, including those to emerging targeted therapies and immunotherapeutic approaches. While early investigations encountered challenges related to intra- and inter-individual variability in ncRNA expression, recent methodological advances have substantially mitigated these limitations, enabling numerous discoveries to progress toward clinical implementation [5].

In the current era of personalized medicine, elucidating the multifaceted roles of ncRNAs in health and disease represents a research imperative of paramount importance. This Special Issue, titled “Circulating Non-Coding RNAs as Biomarkers of Human Diseases,” features eight original research articles and five reviews that collectively advance our understanding of the diagnostic, prognostic, and mechanistic significance of c-ncRNAs in clinical practice. Below is an overview of the contributions included in this Special Issue.

The article by Escuin et al. (contribution 1) provides a proof-of-principle study comparing miRNA expression profiles across matched plasma, tumor tissue, and sentinel lymph node (SLN) samples from 30 estrogen-receptor-positive early breast cancer patients. The authors identified a striking inverse relationship between circulating and tissue miRNAs, with most plasma miRNAs showing downregulation. Importantly, miR-642a-3p and miR-223 were found to be upregulated in patients with metastatic SLNs. Gene ontology and pathway enrichment analyses linked these miRNAs to processes central to metastasis, including epithelial–mesenchymal transition, epithelial cell proliferation, and regulation of receptor tyrosine kinase. These findings highlight the complexity of interpreting circulating miRNA (c-miRNAs) signatures and underscore the potential of specific plasma miRNAs as surrogate markers for lymph node involvement in early breast cancer.

In the second article, Córdoba-Lanús et al. (contribution 2) present a longitudinal study assessing c-miRNAs as early biomarkers of lung cancer (LC) in patients with chronic obstructive pulmonary disease (COPD). Over six years of follow-up, four miRNAs (miR-1246, miR-206, miR-224-5p, and miR-194-5p) were found to be dysregulated in COPD patients who later developed LC. Validation in an independent cohort confirmed altered expression of both miR-1246 and miR-206 up to three years before LC diagnosis, implicating their role in cancer-related pathways. Notably, miR-206 dysregulation was evident well before clinical onset, highlighting its promise as a predictive biomarker for early identification of high-risk COPD patients and enabling targeted screening and intervention.

The article by Gil-Martínez et al. (contribution 3) examines two inflammation-related miRNAs—hsa-miR-26a-1-3p and hsa-miR-376a-3p—in asthma phenotypes and obesity. Elevated levels of miR-26a-1-3p and reduced levels of miR-376a-3p were observed in obese asthma patients, with both linked to key pathogenic pathways including p53 signaling and extracellular matrix interactions. Validation of target genes in lung tissues further supported their functional relevance. These findings suggest that miR-26a-1-3p and miR-376a-3p hold promise as biomarkers for asthma phenotyping, particularly in distinguishing eosinophilic inflammation and obesity-associated disease, with potential implications for personalized therapy.

The fourth article by Ai et al. (contribution 4) investigates the role of circTTN, a circular intronic RNA, in skeletal muscle development, with particular focus on myoblast proliferation and differentiation. In C2C12 myoblasts, circTTN was shown to act as a negative regulator of myogenesis, as its overexpression suppressed both proliferation and differentiation. Mechanistically, circTTN was shown to recruit the Pur-beta (PURB) protein to the promoter region of its host gene, Titin (TTN), thereby repressing TTN transcription. As PURB is itself an inhibitor of myoblast growth, the circTTN–PURB interaction establishes a regulatory axis that amplifies this inhibitory effect. These findings reveal a previously unrecognized mechanism by which circular RNAs can regulate host gene expression and muscle development, providing new insights into circRNA-mediated transcriptional control with potential implications for muscle disorders and regenerative medicine.

The work by Ng et al. (contribution 5) evaluates serum miRNAs as potential biomarkers for non-alcoholic fatty liver disease (NAFLD) in patients with colorectal polyps. By analyzing expression ratios of eight candidate miRNAs, the authors developed a diagnostic panel that improved markedly in accuracy when patients with other metabolic disorders were excluded (AUC = 0.8337). These findings highlight the promise of c-miRNA ratios for early NAFLD detection in high-risk populations and their potential role in guiding screening strategies.

The sixth article by, Iwańczyk et al. (contribution 6), examines the diagnostic potential of c-miRNAs in coronary artery aneurysmal disease (CAAD). In a cohort of 105 patients stratified into CAAD, coronary artery disease (CAD), and normal coronary artery (NCA) groups, the authors validated candidate miRNAs identified in a previous work by their group [6]. Among them, miR-451a and miR-328-3p demonstrated the most substantial diagnostic value, with miR-451a elevated in CAAD versus CAD and miR-328-3p increased in CAAD compared with NCA. Notably, integrating these biomarkers into conventional risk models significantly improved diagnostic accuracy. These findings highlight the promise of miR-451a and miR-328-3p as non-invasive tools for distinguishing CAAD from related coronary conditions.

Nopp et al.’s work (contribution 7) is a multi-phase study evaluating c-miRNAs as prognostic biomarkers for major adverse cardiovascular events (MACEs) in atrial fibrillation (AF). Within a prospective registry of 347 patients, small RNA sequencing and staged validation identified miR-411-5p as consistently associated with cardiovascular mortality and adverse outcomes. These findings position c-miR-411-5p as a promising non-invasive biomarker for risk stratification in AF, with potential to support more personalized approaches to cardiovascular care.

The article by Avgeros et al. (contribution 8) examines plasma miR-146a and miR-155 as non-invasive biomarkers for Mycosis Fungoides (MFs), the most common cutaneous T-cell lymphoma, and studied the influence of genetic variants on their expression. Both miRNAs were elevated in MF patients, including early-stage cases, and showed higher levels in advanced disease, with miR-155 notably increased in patients with skin tumors or erythroderma. SNP analysis revealed associations between specific genotypes and altered miRNA expression, linking genetic variation to MF susceptibility. These findings highlight miR-146a and miR-155 as promising biomarkers for MF diagnosis and staging, while underscoring the role of host genetics in modulating disease risk.

The article by Beňačka et al. (contribution 9) is a review that provides a broad overview of ncRNAs—including miRNAs, piRNAs, lncRNAs, and others—as emerging diagnostic, prognostic, and therapeutic targets. Beyond their fundamental roles in regulating gene expression and cellular processes, ncRNAs are implicated in cancer, cardiovascular, neurodegenerative, and autoimmune diseases. The authors emphasize that ncRNAs, owing to their stability and disease-specific signatures, represent attractive liquid biopsy markers, while therapeutic approaches such as antisense oligonucleotides, RNA mimics, and CRISPR-based tools further underscore their potential in precision medicine.

The tenth article in this Special Issue, a review by Felekkis et al. (contribution 10), examines the potential of c-miRNAs as biomarkers in osteoarthritis (OA), with emphasis on knee disease. Key candidates such as miR-146a, miR-21, and miR-140 are involved in inflammation, cartilage degradation, and chondrogenesis, and their stability in extracellular vesicles makes them attractive for non-invasive testing. While promising for early diagnosis, disease monitoring, and personalized therapy, the authors highlight persistent challenges—including technical variability and the need for large-scale validation—that must be addressed to translate c-miRNAs into clinical practice.

Dzamar et al.’s study (contribution 11) is a systematic review on the role of miRNAs and exosomal miRNAs in cholesteatoma, a destructive cystic lesion of the temporal bone characterized by abnormal keratinocyte proliferation and bone resorption. Screening four major databases, the authors identified 18 studies reporting consistent dysregulation of multiple miRNAs, including downregulation of exosomal miR-17, miR-10a-5p, miR-125b, and miR-34a, and upregulation of exosomal miR-106b-5p, miR-21-3p, and miR-199a, among others. These miRNAs regulate key processes such as proliferation, apoptosis, differentiation, and tissue remodeling, underscoring their dual potential as biomarkers and therapeutic targets. The review also highlights the unique role of exosomes as carriers of pathogenic miRNAs and as promising vehicles for future treatment strategies.

The review article by Kierbiedź-Guzik and Sozańska (contribution 12) discusses recent advances in understanding the role of miRNAs as biomarkers for asthma therapy, emphasizing their potential to enable personalized clinical management. The authors discuss how specific miRNAs can predict treatment response, detect early exacerbations, and monitor patient compliance across therapies ranging from glucocorticosteroids and β-mimetics to biologics. Given their regulatory role in cytokine production, immune modulation, airway remodeling, and inflammation, miRNA expression profiles are closely tied to disease severity and therapeutic outcomes. By mapping miRNA-driven molecular pathways implicated in asthma, the review underscores opportunities for precise therapeutic targeting and individualized treatment strategies. While promising, the authors stress that further validation is needed to fully integrate miRNA-based tools into routine asthma care.

The article by Pieri et al. (contribution 13) is a review that explores how cardio-specific miRNAs become deregulated after SARS-CoV-2 infection and examines their potential role in the development of cardiovascular diseases. Altered expression levels of c-miRNAs such as miR-146a, miR-27a-5p, miR-21, and miR-133a have been linked to cardiac remodeling, arrhythmias, and atherosclerosis in post-COVID-19 patients. The authors propose that viral-driven inflammation and immune dysregulation may trigger miRNA-mediated pathways leading to cardiovascular damage, underscoring the need for further studies to validate these findings and explore their biomarker and therapeutic potential.

In summary, the articles in this Special Issue highlight the versatility and clinical promise of c-ncRNAs as biomarkers across cancer, cardiovascular, metabolic, and inflammatory diseases. From predicting lung cancer years before diagnosis to stratifying cardiovascular risk and refining disease phenotyping, these studies illustrate the power of ncRNAs to inform diagnosis, prognosis, and therapeutic monitoring. While challenges in standardization and clinical translation remain, the convergence of technological advances, mechanistic insights, and growing clinical evidence positions c-ncRNAs at the forefront of precision medicine, with the potential to transform patient care through improved diagnostic accuracy, prognostic precision, and personalized therapy.

## Data Availability

No new data was created in this study.
